# Protective Effects of *Arbutus unedo* L. Honey in the Alleviation of Irinotecan-Induced Cytogenetic Damage in Human Lymphocytes—An In Vitro Study

**DOI:** 10.3390/ijms24031903

**Published:** 2023-01-18

**Authors:** Andreja Jurič, Irena Brčić Karačonji, Uroš Gašić, Dušanka Milojković Opsenica, Saša Prđun, Dragan Bubalo, Dražen Lušić, Nada Vahčić, Nevenka Kopjar

**Affiliations:** 1 Institute for Medical Research and Occupational Health, Ksaverska Cesta 2, 10000 Zagreb, Croatia; 2 Faculty of Health Studies, University of Rijeka, Viktora Cara Emina 5, 51000 Rijeka, Croatia; 3 Institute for Biological Research “Siniša Stanković”—National Institute of Republic of Serbia, University of Belgrade, Bulevar Despota Stefana 142, 11060 Belgrade, Serbia; 4 Faculty of Chemistry, University of Belgrade, Studentski trg 12-16, 11158 Belgrade, Serbia; 5 Faculty of Agriculture, University of Zagreb, Svetošimunska Cesta 25, 10000 Zagreb, Croatia; 6 Faculty of Medicine, University of Rijeka, Braće Branchetta 20, 51000 Rijeka, Croatia; 7 Faculty of Food Technology and Biotechnology, University of Zagreb, Pierottijeva 6, 10000 Zagreb, Croatia

**Keywords:** chromosome aberration analysis, *Arbutus unedo*, cytokinesis-block micronucleus cytome assay, honey, irinotecan, lymphocytes, phenolics, UHPLC-LTQ Orbitrap MS

## Abstract

Strawberry tree (*Arbutus unedo* L.) honey (STH) has been used since ancient times as a folk medicine remedy, especially in certain Mediterranean countries. This honey, rich in phenolic content, is well recognized for its antioxidant, anti-inflammatory, and antimicrobial activities, and is used for the treatment of skin lesions as well as gastrointestinal and respiratory disorders. This study investigated whether STH alleviates genome damage in human peripheral blood lymphocytes produced by the cytotoxic drug irinotecan. The phenolic profile of STH was previously estimated by ultra-high-performance liquid chromatography coupled to a linear ion trap–Orbitrap hybrid mass spectrometer. The effects of STH were evaluated at three concentrations (1×, 5×, and 10×), based on the daily consumption of the honey by an adult person. After 2 h of in vitro exposure, standard lymphocyte cultures for the analysis of chromosome aberrations and the cytokinesis-block micronucleus cytome assay were established. Our results demonstrate that STH offered remarkable geno- and cytoprotection when administered with irinotecan. These findings are relevant for drawing preliminary conclusions regarding the in vitro safety of the tested honey. However, further studies are needed with the application of more complex experimental models.

## 1. Introduction

Despite the widespread consumption of various types of honey, there is a general deficiency of information regarding the nature and mechanisms beyond many of their protective effects, in particular, at the genome and cell level. This is especially true for some rare types of honey whose productions are limited to certain geographical regions.

Strawberry tree (*Arbutus unedo* L.) honey (STH) has long been recognized as a folk medicine remedy, especially in certain Mediterranean countries [1]. Existing data on the phytochemical composition and biological properties of STH suggest that this type of honey, owing to the high amounts of phenolic compounds, possesses a strong antioxidative activity [2,3,4]. Among the many phenolic constituents of STH, the outstanding one is homogentisic acid (HGA; 2,5-dihydroxyphenylacetic acid), which is a chemical marker of the botanical origin of this honey [5]. As reported by Rosa et al. [6], this compound significantly contributes to the STH antioxidant activity, and exerts a remarkable in vitro protective effect on epithelial colorectal adenocarcinoma (Caco-2) cells. Our recent study proposed that in vitro exposure of human peripheral blood lymphocytes to HGA reduced the damage caused by cytotoxic agent irinotecan (IRI) [7]. Afrin et al. [8] reported on the potential chemopreventive action of STH from Sardinia, Italy. They found that STH induced growth inhibition of human cancer-derived cells HCT-116 and LoVo, along with increased intracellular generation of reactive oxygen species (ROS). The findings obtained in vitro [9] indicate the low cytotoxicity of STH, its phenolic extract, and HGA in three tumor-derived cell lines (HepG2, CAL 27, and Caco-2) without concentration- or time-dependent impacts on cell survival. It was also observed that STH, even at the highest concentrations, did not significantly enhance ROS production compared to the control, irrespective of the cell line and duration of treatment. Interestingly, phenolic extract and HGA were even more efficient in diminishing ROS formation than STH itself. Despite the results obtained from tumor cell lines, evidence of STH effects upon normal (healthy) cells or regular physiological processes is still lacking. For several years, our research team has been devoted to an extensive study of biological and chemical properties of this honey. Brčić Karačonji et al. [10] studied the beneficial effects of short-term consumption of STH in healthy male volunteers, and documented that it increased serum iron levels, lowered liver enzyme activity, as well as augmented white blood cell and thrombocyte counts. Jurič et al. [11] found that consumption of STH by healthy subjects decreased the sensitivity of their leukocyte DNA towards hydrogen-peroxide-related damage. Furthermore, the results of our recent in vitro study on UVB-exposed human lymphocytes [12] confirmed the antioxidative and protective effects of STH.

The encouraging results obtained during our forgoing research have motivated us to propose the hypothesis that some of the mechanisms beyond STH action originate from geno- and cytoprotective effects. With regard to the STH safety profile, this in vitro study was designed to prove (1) whether STH is non-harmful, non-toxic, and biologically compatible with human cells, and (2) whether STH could weaken the toxic effects of IRI, a cytotoxic agent widely administered in chemotherapy against colorectal cancers. As a simple cell model, we chose human peripheral blood lymphocytes along with two common cytogenetic methods, chromosomal aberration (CA) assay and cytokinesis-block micronucleus (CBMN) assay, using a cytome approach. While structural CAs are recorded in the first metaphase following phytohemagglutinin stimulation [13], the cytogenetic damage detectable by CBMN assay requires one more cell division to be expressed [14].

We expect that this research will add new data useful for further explanation of the biological activity of STH and indicate directions for the upcoming related experiments with this type of honey.

## 2. Results

### 2.1. Assessment of Genotoxic and Cytotoxic Properties of Strawberry Tree Honey (STH)

#### 2.1.1. Chromosome Damage

**Figure 1:**
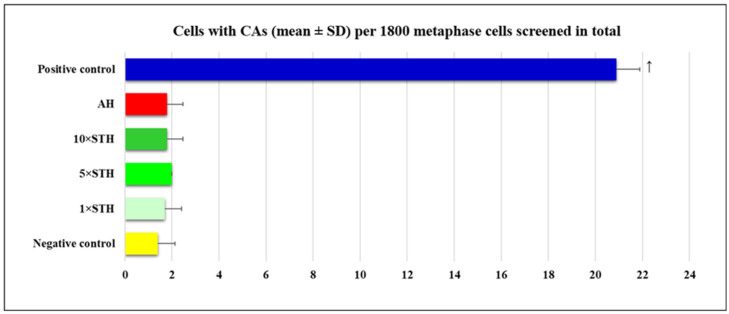
Incidence of cells with CAs observed after treatment with strawberry tree honey (STH) and artificial honey (AH). Negative control (non-treated cells) and positive control (bleomycin—15 mg/L) groups were studied in parallel. Significantly increased value (*p* < 0.05) is ↑—vs. all other experimental groups.

Results obtained by CA assay are shown in Figure 1. Significantly increased incidence of cells with CAs was found only in the positive control.

As reported in Table 1, the positive control sample showed the highest CA count. At the concentrations tested, STH did not produce considerable chromosome breakage effects. Similar conclusions can be applied to AH.

#### 2.1.2. Cytokinesis-Block Micronucleus Cytome (CBMN) Assay

The findings of the CBMN assay are shown in Table 2. The highest levels of micronuclei (MNi), nuclear buds (NBs), and nucleoplasmic bridges (NPBs) were observed in the positive control. None of the other treatments resulted in the formation of NPBs. STH did not show a significant ability to produce MNi or NBs. Treatment with AH led to a higher production of MNi and NBs in relation to the negative control and all STH-treated samples. However, the only statistically significant difference was the number of NBs, which deviated from the negative control.

Parameters related to lymphocyte proliferation are reported in Table 3. The lowest nuclear division index (NDI) value was found in the positive control sample. Higher scores of mononucleated cells (M1) in STH-treated cells caused a minor concentration-dependent decrease in NDI compared to the negative control. However, the only statistically significant difference was observed between the 1 × STH-treated sample and the negative control. Treatment with AH significantly lowered the NDI compared to all STH-treated samples and the negative control.

The cytome approach of the CBMN assay enables the simultaneous determination of non-viable cells on the same microscope preparations, owing to the morphological features typical of apoptosis or necrosis. We found that all of our treatments resulted in increased proportions of non-viable cells in relation to the negative control. In all samples, apoptosis prevailed over necrosis. The highest incidence of lymphocyte death was observed in the positive control (Table 4). AH treatment showed higher cytotoxic potential compared to STH.

Treatment-related cytostatic effects were proven by estimating the cytokinesis-block proliferation index (CBPI). The observed range of CBPI values was positive control < AH < STH < negative control (Table 4).

### 2.2. Assessment of Genoprotective and Cytoprotective Properties of STH against IRI-Induced Cytogenetic Damage

#### 2.2.1. Chromosomal Aberration (CA) Assay

**Figure 2:**
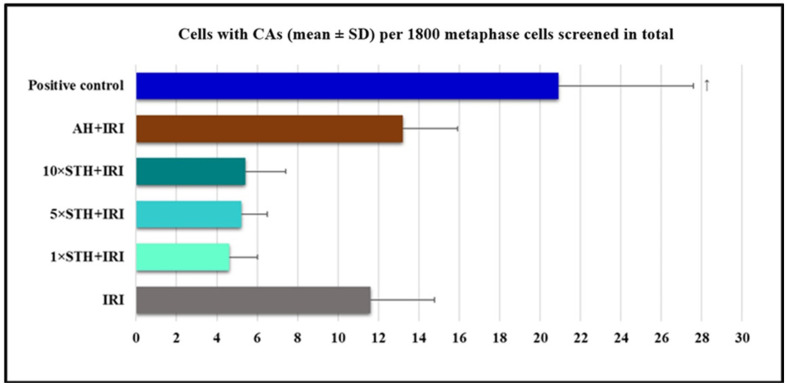
Frequency of cells according to CA assay after treatment with different combinations of cytotoxic drug irinotecan (IRI) with strawberry tree honey (STH) and artificial honey (AH). Single-IRI-treated cells and positive control (bleomycin—15 mg/L) cells were studied in parallel. Significantly increased (*p* < 0.05) value is ↑—vs. all other experimental groups.

Figure 2 displays findings on the frequency of cells with CAs when treated with a single IRI, IRI administered together with different STH concentrations, IRI administered together with AH, and in the positive control.

The highest number of cells with CAs was found in the positive control, which significantly differed from all of the other experimental groups. Combined treatment with AH and IRI also caused a high yield of cells with CAs, slightly larger than IRI in isolation. STH, when administered in combination, counteracted the cytogenetic damage produced by IRI and significantly lowered the incidence of cells with CAs (Figure 2).

From the values reported in Table 5, one can see that bleomycin, used as positive control, caused more structural CAs than single IRI and all of the combined treatments. STH lowered the production of chromatid breaks and acentric fragments compared to IRI. In contrast, AH did not offer protection against IRI-induced cytogenetic damage.

#### 2.2.2. Cytokinesis-Block Micronucleus Cytome (CBMN) Assay

Table 6 reports the findings regarding the incidence of MNi, NBs, and NPBs in treated and positive control samples. The most prominent damage was in the range positive control > combination of AH with IRI > single IRI. STH offered marked protection against IRI-induced damage in terms of MNi and NB formation. Additionally, in combined treatments with STH, no NPBs were recorded.

**Figure 3:**
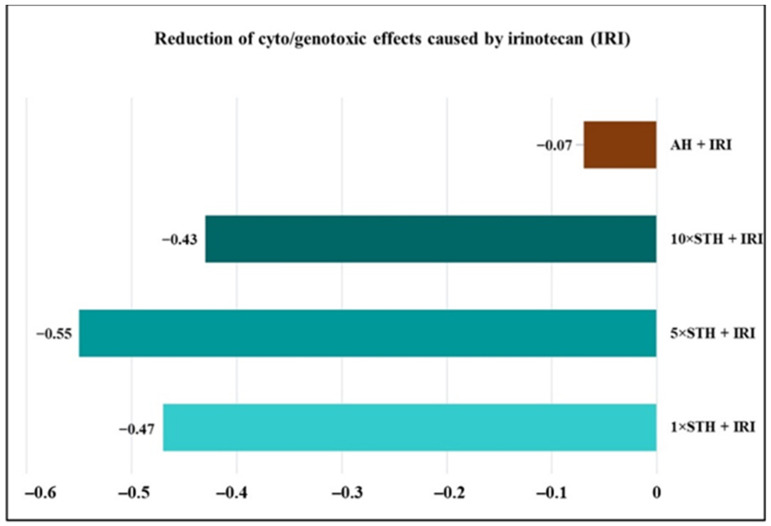
Influence of strawberry tree honey (STH), artificial honey (AH), and strawberry tree honey phenolic extract (E) on the reduction in cyto/genotoxic effects caused by irinotecan (IRI).

To establish whether STH can alleviate the damaging potential of IRI when administered in combination, we determined an additional index for each culture. In the first step, we looked for the cut-off value of this index, considering the total number of MNi in the IRI-treated sample. We found that the number of MNi that corresponded to a decline at 5% significance level amounted to 88, while an increase would be achieved at 150 MNi. Based on these values, we found the cut-off for a decline in MNi frequency to be −0.248, and for its rise the cut-off was 0.282. Then, we set out to find values of the index for all combinations of tested compounds and IRI, and related them to the cut-offs. Since all of the obtained index values for treatments with STH were above the cut-off (−0.248), we conclude that STH, when applied in combination, efficiently diminished the toxic and harmful cytogenetic effects produced by IRI. Artificial honey was not effective in this regard (Figure 3).

**Figure 4:**
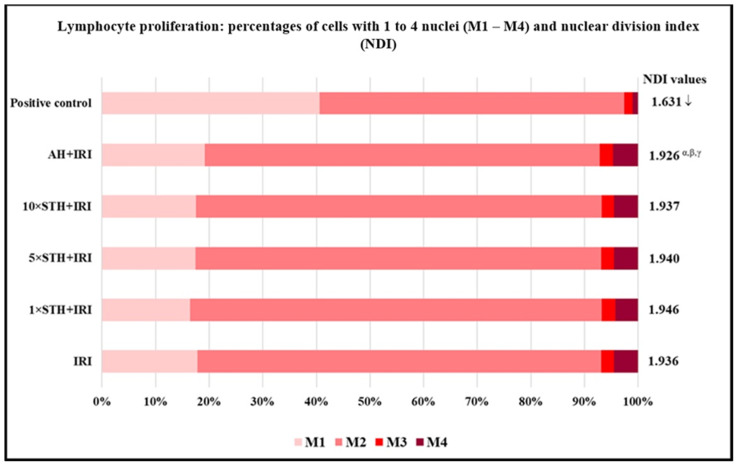
Parameters of cell proliferation established after treatment with different combinations of cytotoxic drug irinotecan (IRI) with strawberry tree honey (STH) and artificial honey (AH). Single-IRI-treated cells and positive control (bleomycin—15 mg/L) cells were studied in parallel. Significantly decreased (*p* < 0.05) values are ↓—vs. all other experimental groups; I—vs. IRI; α—vs. 1 × STH + IRI sample; β—vs. 5 × STH + IRI sample; γ—vs. 10 × STH + IRI sample.

Figure 4 shows the results regarding parameters related to lymphocyte proliferation. The lowest NDI value was found in the positive control sample. The NDI value after combined treatment with IRI and AH was slightly lower compared to single IRI. In contrast, combined treatments with STH caused a minor (but non-significant) rise in NDI value compared to the IRI sample.

**Figure 5:**
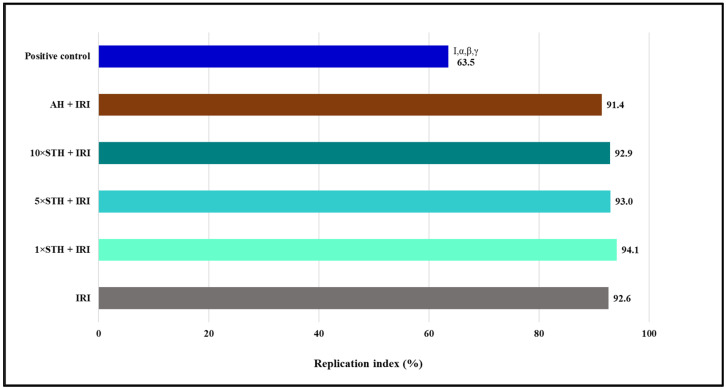
Values of replication index established after treatment with different combinations of the cytotoxic drug irinotecan (IRI) with strawberry tree honey (STH) and artificial honey (AH). Single-IRI-treated cells and positive control (bleomycin—15 mg/L) cells were studied in parallel. Significantly decreased (*p* < 0.05) values are I—vs. IRI; α—vs. 1 × STH + IRI sample; β—vs. 5 × STH + IRI sample; γ—vs. 10 × STH + IRI sample.

Figure 5 shows the obtained values of the RI. The greatest reduction in lymphocyte proliferation was observed in the positive control.

**Figure 6:**
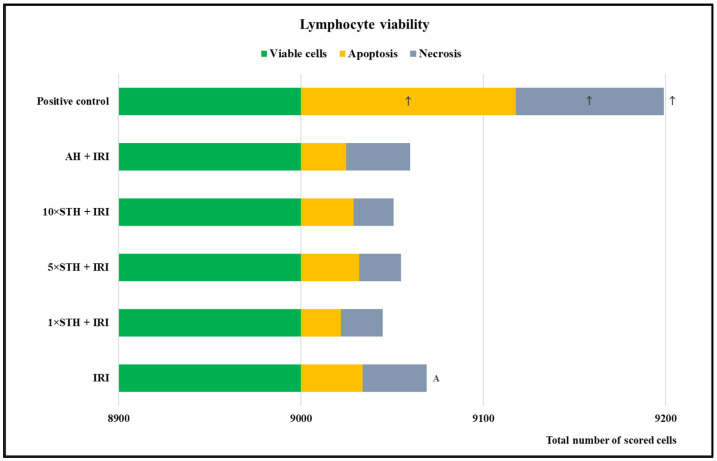
Cell viability effects established after treatment with different combinations of the cytotoxic drug irinotecan (IRI) with strawberry tree honey (STH) and artificial honey (AH). Single-IRI-treated cells and positive control (bleomycin—15 mg/L) cells were studied in parallel. The marks placed inside the categories on the bars refer to the distinct form of cell death (apoptosis or necrosis), while those placed outside the bars denote the total number of non-viable cells. Significantly increased (*p* < 0.05) values are ↑—vs. all other experimental groups; A—vs. 1 × STH + IRI sample.

The results regarding lymphocyte viability are shown in Figure 6. The bleomycin-treated sample (positive control) had the highest proportion of non-viable cells. STH efficiently counteracted IRI effects, causing lower yields of non-viable cells.

**Figure 7:**
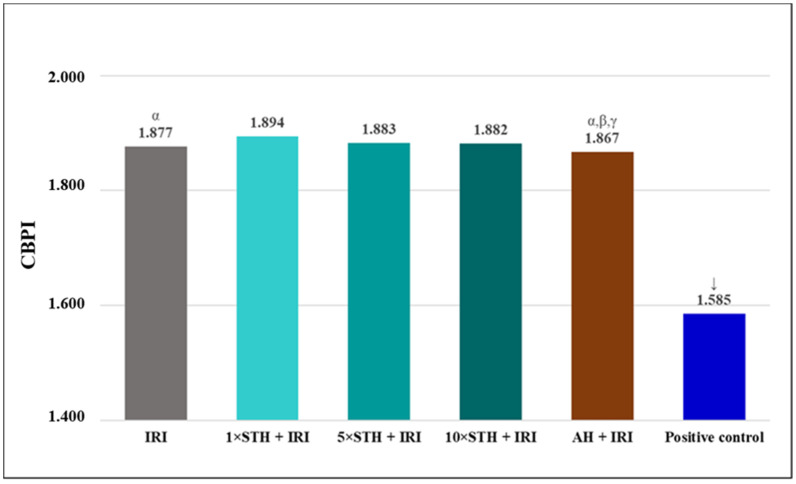
Values of cytokinesis-block proliferation index (CBPI) established after treatment with different combinations of the cytotoxic drug irinotecan (IRI) with strawberry tree honey (STH) and artificial honey (AH). Single-IRI-treated cells and positive control (bleomycin—15 mg/L) cells were studied in parallel. Significantly decreased (*p* < 0.05) values are α—vs. 1 × STH + IRI sample; β—vs. 5 × STH + IRI sample; γ—vs. 10 × STH + IRI sample, ↓—vs. all other experimental groups.

To estimate the extent of cytostatic effects produced by treatments, the CBPI was calculated for each experimental group. The results are reported in Figure 7. The lowest CBPI value was produced by treatment with bleomycin, used as the positive control. When compared to a single IRI, combined treatments with STH caused a slight increase in the CBPI (Figure 7).

**Figure 8:**
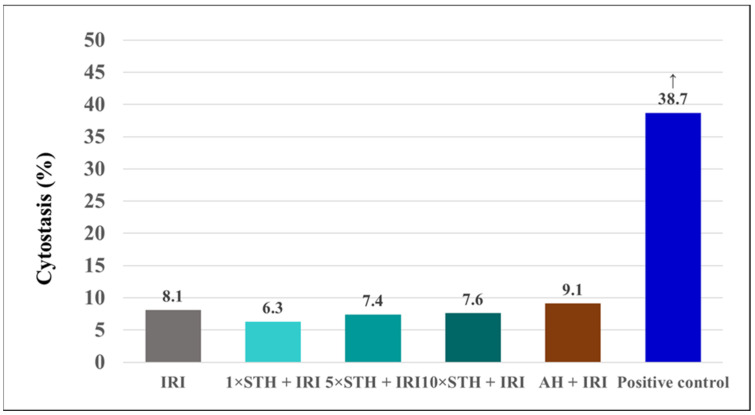
The extent of cytostasis produced in the cultures established after treatment with different combinations of the cytotoxic drug irinotecan (IRI) with strawberry tree honey (STH) and artificial honey (AH). Single-IRI-treated cells and positive control (bleomycin—15 mg/L) cells were studied in parallel. Significantly increased (*p* < 0.05) value is ↑—vs. all other experimental groups.

Figure 8 shows the results regarding the extent of cytostasis produced in the cultures of human peripheral blood lymphocytes; cytostasis was the highest in the positive control.

## 3. Discussion

Not many studies have, up to now, investigated the influence of honey at the genome and cell level. In 2017, Yaacob et al. [15] published an extensive review that provides good insight into the existing studies that investigated the association between honey and genomic stability. Evidently, there is no specific rationale for investigating and establishing the protective effects for well-known honey types that humankind has used since ancient times. However, there are some relatively rare, locally important types of honey, such as STH, potentially valuable in the development of novel nutraceuticals, whose production is limited to particular geographical areas and whose phytochemical, biochemical, and biological effects, in spite of a long tradition of use, are still poorly documented.

The present study is the first to assess and confirm that STH exerts a high biocompatibility and remarkable protective effects in human lymphocytes treated with IRI. As established in many preceding studies with other natural products, such effects are largely associated with the antioxidative potential of the tested substances. Existing reports suggest that high concentrations of phenolic compounds, especially HGA, contribute to superior antioxidative properties of STH compared with other types of honey [2,3,6,16,17].

We propose that the greatest protective potential of STH on the lymphocyte model implemented in the present study was also achieved by HGA, the concentration of which was rather remarkable, i.e., 306.83 mg/kg. The second abundant bioactive component in our STH sample was acacetin. Since its content was higher than other phenolic constituents, it is possible that acacetin also made a great contribution in terms of cyto/geno protection. This statement, however, has to be proven in forthcoming studies. Up to now, the effects of acacetin, especially at the cellular level, have been relatively poorly investigated, and, in the available literature, there are no comparable data on studies that use the same model and methodological approach as in our research.

Additional to HGA and acacetin, in our STH, there were relatively high amounts of *p*-hydroxybenzoic acid, *p*-hydroxyphenylacetic acid, and *p*-coumaric acid. Other compounds present in lower amounts were gallic acid, ferulic acid, caffeic acid, protocatechuic acid, quercetin, pinocembrin, apigenin, and chrysin. When discussing the effects of a natural product such as honey, it has to be noted that they usually depend not only on its qualitative and quantitative composition, but also on the complex interactions between the present components. Many of the mentioned phenolic constituents exert a “dual nature” (pro-oxidant and antioxidant properties) [7,18,19,20]. As known, their pro-oxidant behavior largely depends on the specific conditions, and is influenced by molecular oxygen and the presence of copper and/or iron ions, which potentiate DNA oxidative damage [19,21,22]. In the complex matrices, however, there is also a possibility that the pro-oxidative effects and genotoxicity of one compound might be modulated by the protective properties of another.

Based on the obtained results, the STH tested in our study showed low cytotoxicity and genotoxicity on the lymphocyte model. The overall low net effects we observed by CA analysis and the CBMN assay obviously resulted from mutual interactions of the potent bioactive compounds contained in the STH, whose biological effects were well established in the previous studies [23,24,25,26,27,28,29,30]. In the present study, a high degree of concordance between the two cytogenetic methods was also found. Generally, high amounts of structural chromosomal aberrations corresponded to a higher incidence of MNi and other features detectable by CBMN assay [31]. Nevertheless, complete results suggest a high biocompatibility of STH with the cell model used in this study.

Beneficial effects of the tested STH on lymphocytes could be also related to the monosaccharide content. Our STH contained 34.2 g fructose and 32.5 g glucose per 100 g of honey. These monosaccharides are “reducing sugars”, which, by donating electrons to other molecules, provide the reducing power needed to diminish oxidative stress. Since both glucose and fructose easily enter into cells, it is likely that STH-treated lymphocytes stored an extra amount of reducing sugars. It may be assumed that the lower geno/cytotoxic effects observed in STH-treated cells could be associated with the potency of these reducing sugars to neutralize oxidative species formed during in vitro cell growth, which in turn contributed to the lower level of DNA and chromosomal damage.

Based on the score of non-viable cells, it is evident that our tested sample of STH possessed low cytotoxic potency. The phenomenon of cytotoxic effects exerted by various types of honey has been well recognized and reviewed [32,33]. Recently, Imtara et al. [34] observed the cytotoxic effects of some Moroccan and Palestinian honeys on human cell lines HCT-116 and MCF-7. Sardinian STH produced cytotoxic effects in cell lines HCT-116 and LoVo [8], showing better anticancer power than that obtained with manuka honey. However, our previous study using the same STH sample [9] indicated high cell viability of CAL27, HepG2, and Caco-2 cell lines when treated with STH, its phenolic extract, and HGA.

In addition to the cytogenetic and cytotoxic effects observed after treatment, we also have to briefly mention the effects of treatments on lymphocyte proliferation. Taken together, STH did not significantly impair lymphocyte proliferation. This is noticeable when examining the values of different indices that describe lymphocyte proliferation (NDI, CBPI, RI), as well the percentage of cytostasis, calculated for the treated lymphocyte cultures. From all of these values, it is clear that STH-treated cells showed more or less normal progression through cell cycles. For the cultivation of lymphocytes, the RPMI-1640 medium was used, in which glucose (contained in 2 g/L) represents a major energy source for growing cells. We assume that additional amounts of fructose and glucose, which easily entered into STH-treated cells, could be utilized as additional sources of energy needed to sustain cell growth and proliferation in vitro. This is the possible reason why STH-treated lymphocytes did not show cell-cycle delays.

The existing literature reports the antiproliferative properties of various types of honey [8,32,33,34,35], as well as antiproliferative and cytostatic effects caused after exposure to the same or related phenolics as those present in our tested sample. Almost all of the abovementioned papers that report on cytotoxic or apoptotic effects of specific phenolic compounds also point to their antiproliferative properties [36,37,38,39,40,41,42,43,44,45,46].

An important aim of this research was to evaluate whether STH could alleviate the cytogenetic damage caused by the antineoplastic drug IRI. Before this study, only one other study [47] actually used a human lymphocyte model in pursuit of potential protective effects of honey at the chromosome level in vitro. The authors demonstrated that the addition of honey significantly lowered mitomycin-C-induced chromosomal damage in lymphocytes of Fanconi anemia patients. However, no information regarding the type of honey and its phytochemical profile was provided, which certainly represents a major weakness of the study. In contrast, we used a honey sample that was previously well characterized, and combined it with an antineoplastic drug that today has an important role in chemotherapy approaches for the treatment of metastatic colorectal cancer [48,49,50]. IRI acts specifically at the DNA level via direct action on DNA topoisomerase I enzymes as well by promoting oxidative stress [51,52,53,54]. Results of our former study [55] indicated that it produces a high level of chromosomal instability in human lymphocytes at concentrations equal to the two most commonly administered therapeutic doses of the drug. In the present study, we used the concentration matched to the dose of IRI 350 mg/m^2^ administered as monotherapy to colorectal cancer patients [56]. The results of our in vitro trials with combined treatments comprising irinotecan, STH, and its phenolic extract demonstrate that both bioactive mixtures offered remarkable geno- and cytoprotection against the antineoplastic drug.

We are aware that our study has certain limitations. Using an in vitro approach, we evaluated the effects of STH on one specific cell type. Direct exposure of cells to the tested compounds, as was the case here, resembles mostly the cases where honey or its derivatives are used in topical treatment, for instance, wound treatment. However, in a real-life situation, honey is more often taken via the oral route. In this case, before it exerts any pharmacological action, honey is subjected to digestion, which causes differences in absorption that result in the different bioavailability of its constituents to various cell types. No matter how informative, the observations resulting from the use of a single-cell model cannot be generalized. At this initial phase of the research, our primary goal was to communicate novel evidence, but not to provide clinically useful information. Furthermore, the present investigation led to many open questions that deserve further research, for instance, whether and how differences in the exposure route affect the protective effects offered by STH, and which responses could be expected in other cell types. Another important issue is that of standardization, considering that STHs from different geographical areas may vary in their phytochemical composition, and that the contents of the main phenolic components may vary over the years. Since all of these factors could modify the biological response, these issues must be further studied in more detail.

## 4. Materials and Methods

### 4.1. Chemicals and Reagents

IRI (CAS No. 100286-90-6, in the form of hydrochloride trihydrate salt) was the product of LC Laboratories (Woburn, MA, USA). Prior to use for the treatments, it was dissolved in 0.9% NaCl solution (Croatian Institute for Transfusion Medicine, Zagreb, Croatia).

Bleomycin (CAS No. 11056-06-7, in the form of bleomycin sulfate) was the product of Nippon Kayaku Co., Ltd., Tokyo, Japan. It served as a positive control, and was also dissolved in 0.9% NaCl solution before use.

AH was composed of fructose (Kemika, Zagreb, Croatia), glucose (Sigma-Aldrich, Steinheim, Germany), maltose (Torlak, Belgrade, Serbia), and sucrose (Fluka, St. Gallen, Switzerland). It was freshly prepared by dissolving sugars in 10 mL of distilled water, as follows: 40 mg of fructose + 30 mg of glucose + 8 mg of maltose + 2 mg of sucrose [3].

Unless otherwise specified, other chemicals were obtained from Sigma Chemical Co. (St. Louis, MO, USA).

### 4.2. Strawberry Tree Honey

The STH sample used in this study originated from the apiary location in Vrgorac, Croatia (apiary coordinates 43.20° N 17.37° E). It was representatively sampled, acquired directly from the beekeeper, and stored in airtight glass jars that were kept at 4 °C in the dark until further analysis. This sample showed a particular unifloral correspondence to the STH features, and was consequently chosen for this experiment. The botanical origin of the honey was determined by sensory and melissopalynological evaluations. It was also confirmed by measuring the chemical marker HGA using gas chromatography–mass spectrometry (GC-MS) [5]. Quantification of phenolic compounds in STH was performed by an ultra-high-performance liquid chromatograph (UHPLC) coupled to a linear ion trap–Orbitrap hybrid mass spectrometer (LTQ Orbitrap MS). Detailed results on the phenolic profiling of the tested STH sample were reported in our previous publication [12]. This experiment was included in a broader study on STH, carried out to complete the doctoral thesis of one of the authors.

A short characterization of the tested STH sample is provided in Table S1.

### 4.3. Blood Sampling

Venous blood was taken from three healthy men (subject #1, 32 years; subject #2, 27 years; subject #3, 32 years), all of which signed informed consent forms prior to blood sampling. They were non-smokers, free from exposure to any radiation or genotoxic agents during the last year. Blood was collected into heparinized vacutainers (Becton Dickinson, Franklin Lakes, NJ, USA). Total amount of blood per subject was 40 mL.

### 4.4. Experimental Schedule

We conducted three independent experiments, using blood samples of each donor, according to the schedule presented in Table 7.

Decision on the lowest tested concentration of STH (1 × STH) relied on the intake of 50 g of the STH/day by an adult person weighing 70 kg (0.715 g of STH/kg of body mass). To search for possible cytogenetic effects, it was proposed that we additionally evaluate 5× and 10× higher concentrations.

IRI was tested at a concentration that matched the dose of 350 mg/m^2^ of drug administered as monotherapy to colorectal cancer patients [56].

Bleomycin concentration was deduced from our earlier in vitro experiments on the same cell model [57,58].

Treatments were performed as follows: blood samples (V = 1200 μL per donor–experimental point–method) were pipetted in sterile Falcon^®^ tubes and mixed with appropriate volumes of the tested compounds. The treatments lasted for 2 h at 37 °C (Heraeus Hera Cell 240 incubator, Langenselbold, Germany). Non-treated blood samples were maintained in identical settings. When the exposure period ended, cell cultures were set up, consistent with standard cytogenetic protocols.

### 4.5. Chromosomal Aberration (CA) Assay

Protocol used for the CA assay adhered the International Programme on Chemical Safety guidelines [13], and the cytogenetic biodosimetry manual issued by the International Atomic Energy Agency [59].

In each independent trial, two matching cultures for every experimental group, including controls, were established. The treated blood (V = 600 μL per culture) was mixed with a growth medium RPMI-1640 (Gibco, Grand Island, NY, USA) complemented with fetal calf serum (Gibco, Grand Island, NY, USA) and antibiotics (penicillin and streptomycin; Sigma-Aldrich, Steinheim, Germany). Lymphocytes were challenged to proliferation with phytohemagglutinin (Remel, Lenexa, KS, USA). Flasks were kept at 37 °C (Heraeus Hera Cell 240 incubator; Langenselbold, Germany). By adding colhicine, following 45 h of cultivation, the cell cycle was stopped in metaphase. At the 48th hour, the harvesting of cells was performed by centrifugation and subsequent treatment with hypotonic KCl solution (0.075 mol/L). To obtain lymphocyte suspensions, several consecutive fixation steps (methanol and glacial acetic acid, 3:1, *v*/*v*) and following centrifugation stages were completed. Clear lymphocyte suspension was dropped onto cold microscope slides. Dried slides were dyed with Giemsa stain (Sigma-Aldrich, Steinheim, Germany; 5% water solution) for 10 min.

Slides were analyzed at 1000× magnification using a light microscope (Leitz, Oberkochen, Germany). Overall, 600 metaphases (3 × 200) per blood donor, which contain 45 to 47 centromeres, were recorded to establish the specific types of aberrations and their total numbers, along with the proportion of cells bearing any aberration. Structural CAs were identified according to the number of breakage events observed on the sister chromatids. After carrying out all three independent trials, data were united; the final values refer to the mean ± standard deviation (SD) or sum of aberrations obtained for total of 1800 metaphases per experimental group of interest.

### 4.6. Cytokinesis-Block Micronucleus Cytome Assay (CBMN) Assay

We followed the protocol proposed by Fenech and Morley [60] and its later adjustments [61]. Lymphocyte cultures were set up and maintained as described for the CA assay. To block cytokinesis, at the 44th hour, cytochalasin B (6 μg/mL) was added. At the 72nd hour, cultures were harvested by centrifugation, followed by hypotonic treatment (0.075 mol/L KCl) and centrifugation. Then, cells were fixed in several repeating stages with methanol:acetic acid (3:1 *v*/*v*). The final cell suspension was used to prepare microscope slides. After air drying, they were subjected to Giemsa staining (10 min, 5% solution). Slides were analyzed at 1000× magnification using a light microscope (Leitz, Oberkochen, Germany). Identification of MNi, NBs, NPBs, and cells in apoptosis or necrosis was performed by scoring 3 × 1000 BN cells per blood donor and experimental point, in line with the recommendations of Fenech et al. [62] and Fenech [61].

To prove the extent of the protective effects exhibited by the tested compounds, we performed additional evaluations [63]. For all experimental groups, using the formula [(MN_IRI + tested compound_ − MN_IRI_)/MN_IRI_], an index was calculated, the value of which shows a decline (if the value is negative) or a rise (if the value is positive) in the MN frequency, related to the cut-off value determined after treatment with the cytotoxic drug in isolation. To establish the cut-off value, the same formula was applied, where MN_IRI + tested compound_ represents the number of MN that led to a decline/rise at a significance level of 5%.

On the same preparations, we counted cells for the calculation of the nuclear division index [NDI = (M_1_ + 2M_2_ + 3M_3_ + 4M_4_)/N]. M_1_–M_4_ denote numbers of mono-, bi-, tri-, and quadrinuclear cells, while N represents the total number of cells counted [64]. To establish the NDI for a particular experimental group, 9000 M_1_–M_4_ cells ((3 × 1000) × 3 blood donors) in total were counted.

The numbers of bi- and multinucleated cells were then used to calculate the replication index, RI = {[(<No. binucleated cells> + <2 × No. multinucleated cells>)/(Total number of cells in treated culture)]/[(<No. binucleated cells> + <2 × No. multinucleated cells>) ÷ (Total number of cells in control culture)]} × 100 [65].

To evaluate the dynamics of lymphocyte proliferation, we also determined the cytokinesis-block proliferation index, CBPI = [(No. mononucleated cells) + (2 × No. binucleated cells) + (3 × No. multinucleated cells)]/(Total number of cells) [66]. To establish the CBPI for a particular experimental group, a total of 9000 mono-, bi-, and multinuclear cells ((3 × 1000) × 3 blood donors) were counted.

Subsequently, CBPI values for the control (CBPI_C_) and the treated cultures (CBPI_T_) were determined. The percentage of cytostasis was calculated as % Cytostasis = 100 − 100 × [(CBPI_T_ − 1)/(CBPI_C_ − 1)] [66].

### 4.7. Statistical Analysis

Statistica software (Data Science Workbench, version 14. (License No. 14.0.0.15; TIBCO Software Inc. 2020; Palo Alto, CA, USA)) was used to compute basic statistic descriptors (mean and standard deviation), and to perform multiple comparisons between groups (analysis of variance (ANOVA) with post hoc Tukey HSD test). Using Pearson’s χ^2^ test for two-by-two contingency tables (http://vassarstats.net/newcs.html 20 February 2021), we tested the significance of the intergroup differences in the parameters of cell viability and proliferation. The level of statistical significance was fixed at 5%.

## 5. Conclusions

All of the collected data reveal a low genotoxic potential of STH towards human peripheral blood lymphocytes. STH also did not impair in vitro proliferation, and offered good geno/cytoprotection against IRI-induced damage in this cell type. These findings are relevant for drawing preliminary conclusions regarding the in vitro safety of the tested honey, considering that all of the results were gathered in three repeated experiments, and all judgements about the chromosomal damage relied on recording a high number of individual cells in each sample. Before drawing any exhaustive conclusion applicable to human risk assessments, further trials applying more complex experimental models are needed. As this was a preliminary study, the authors are planning an extension of the research. The limitations set by the applied experimental design will certainly be overcome in forthcoming studies.

## Figures and Tables

**Table 1:** Results of CA assay on human lymphocytes after treatment with strawberry tree honey (STH) and artificial honey (AH). Negative control (non-treated cells) and positive control (bleomycin—15 mg/L) groups were studied in parallel.

Group	Chromatid Break	Chromosome Break	Acentric Fragment	Dicentric Chromosome	Total
**Negative control**	10 1.1 ± 0.60	- -	3 0.3 ± 0.50	- -	13 1.4 ± 0.73
**1 × STH**	10 1.1 ± 0.60	- -	5 0.6 ± 0.53	- -	15 1.7 ± 0.71
**5 × STH**	14 1.6 ± 0.53	- -	4 0.4 ± 0.53	- -	18 2.0 ± 0.0
**10 × STH**	12 1.3 ± 0.50	- -	4 0.4 ± 0.53	- -	16 1.8 ± 0.67
**AH**	12 1.3 ± 0.71	- -	4 0.4 ± 0.73	- -	16 1.8 ± 0.67
**Positive control**	157 17.4 ± 7.26 ^↑^	18 2.0 ± 1.50 ^↑^	70 7.8 ± 1.64 ^↑^	11 1.4 ± 0.74 ^↑^	256 28.4 ± 7.33 ^↑^

Data represent the sum (upper row) of CAs per experimental group, and their mean ± SD (lower row) based on nine independent scorings. Significantly increased (*p* < 0.05) value is ↑—vs. all other experimental groups.

**Table 2:** Micronuclei (MNi), nuclear bud (NB), and nucleoplasmic bridge (NPB) formation in human lymphocytes after treatment with strawberry tree honey (STH) and artificial honey (AH). Negative control (non-treated cells) and positive control (bleomycin—15 mg/L) groups were studied in parallel.

Group	Micronuclei (MNi)							Nuclear Buds (NBs)		Nucleoplasmic Bridges (NPBs)	
	Mean (MNi)_1000_ ± SD	Total (MNi)_9000_	Mean (BN_MN_)_1000_ ± SD	Total (BN_MN_)_9000_	Distribution of BN_MN_ Cells with			Mean (NBs)_1000_ ± SD	Total (NBs)_9000_ *	Mean (NPBs)_1000_ ± SD	Total (NPBs)_9000_ *
					1 MN	2 MN	3 MN				
**Negative** **control**	2.4 ± 0.53	22	2.4 ± 0.53	22	22	0	0	1.9 ± 0.60	17	0	0
**1 × STH**	2.7 ± 0.50	24	2.7 ± 0.50	24	24	0	0	2.4 ± 0.53	22	0	0
**5 × STH**	2.8 ± 0.67	25	2.8 ± 0.67	25	25	0	0	2.2 ± 0.83	20	0	0
**10 × STH**	2.3 ± 0.50	21	2.3 ± 0.50	21	21	0	0	2.8 ± 0.44	25	0	0
**AH**	3.1 ± 0.60	28	3.1 ± 0.60	28	28	7	1	3.2 ± 0.83 ^N^	29	0	0
**Positive control**	17.6 ± 2.07 ^↑^	158	15.6 ± 1.81 ^↑^	140	123	16	1	9.8 ± 1.86 ^↑^	88	1.4 ± 0.73 ^↑^	13

Significantly increased (*p* < 0.05) value is ↑—vs. all other experimental groups; N—vs. negative control. *—BN cells contained only a single NB or NPB.

**Table 3:** Parameters of cell proliferation observed after treatment with strawberry tree honey (STH) and artificial honey (AH). Negative control (non-treated cells) and positive control (bleomycin—15 mg/L) groups were studied in parallel.

Group	Parameters of Cell Proliferation					
	Cells with 1 to 4 Nuclei (%)				Nuclear Division Index (NDI)	Replication Index (%)
	M_1_	M_2_	M_3_	M_4_		
**Negative** **control**	15.4	73.5	3.7	7.4	2.031	100
**1 × STH**	17.4	71.4	3.4	7.8	2.015 ^N^	97.8
**5 × STH**	16.3	73.1	3.1	7.5	2.020	98.4
**10 × STH**	15.6	73.3	3.5	7.6	2.030	99.5
**AH**	16.4	74.6	2.9	6.2	1.989 ^N,α,β,γ^	96.7
**Positive control**	40.6	56.8	1.5	1.1	1.631 ^↓^	63.5 ^↓^

Significantly decreased (*p* < 0.05) values are ↓—vs. all other experimental groups; N—vs. negative control; α—vs. 1 × STH sample; β—vs. 5 × STH sample; γ—vs. 10 × STH sample; δ—vs. sample treated with AH.

**Table 4:** Cell viability and cytostatic effects observed after treatment with strawberry tree honey (STH) and artificial honey (AH). Negative control (non-treated cells) and positive control (bleomycin*—*15 mg/L) groups were studied in parallel.

Group	Cell Viability			Cytostatic Effects	
	No. of Cells in Apoptosis	No. of Cells In Necrosis	Total No. of Dead Cells	CBPI	Cytostasis (%)
**Negative control**	13	3	16	1.954	0
**1 × STH**	14	12 *	26 *	1.932 ^#^	2.3
**5 × STH**	20	15 *	35 *	1.937	1.8
**10 × STH**	18	16 *	34 *	1.947	0.7
**AH**	19	16 *	35 *	1.920 ^#,α,β,γ^	3.6
**Positive control**	118 ^↑^	81 ^↑^	199 ^↑^	1.585 ^↓^	38.7 ^↑^

Significantly increased (*p* < 0.05) values are ↑—increased vs. all other experimental groups; *—increased vs. negative control. Significantly decreased values are: ↓—decreased vs. all other experimental groups; #—decreased vs. negative control; α—decreased vs. 1 × STH sample; β—decreased vs. 5 × STH sample; γ—decreased vs. 10 × STH sample.

**Table 5:** Results of CA assay after treatment with different combinations of cytotoxic drug irinotecan (IRI) with strawberry tree honey (STH) and artificial honey (AH). Single-IRI-treated cells and positive control (bleomycin*—*15 mg/L) cells were studied in parallel.

Group	Chromatid Break	Chromosome Break	Acentric Fragment	Dicentric Chromosome	Quadriradial Chromosomes	Total
**IRI**	59 6.6 ± 2.07 ^#^	5 0.6 ± 0.88	46 5.1 ± 2.15 ^#^	4 0.4 ± 0.53 ^α,β^	2 0.2 ± 0.67	116 12.9 ± 3.82 ^#^
**1 × STH + IRI**	18 2.0 ± 0.87	4 0.4 ± 0.53	16 1.8 ± 0.83	- -	5 0.6 ± 0.88	43 4.8 ± 1.30
**5 × STH + IRI**	24 2.7 ± 1.41	5 0.6 ± 0.73	14 1.6 ± 0.88	- -	5 0.6 ± 0.73	48 5.3 ± 1.22
**10 × STH + IRI**	26 2.9 ± 1.54	3 0.3 ± 0.71	17 1.9 ± 0.78	2 0.2 ± 0.44	5 0.6 ± 0.73	53 5.9 ± 1.96
**AH + IRI**	80 8.9 ± 2.98 ^I,α,β,γ^	11 1.2 ± 0.97	35 3.9 ± 2.26 ^α,β^	2 0.2 ± 0.44	1 0.1 ± 0.33	129 14.3 ± 3.43 ^α,β,γ^
**Positive control**	157 17.4 ± 7.26 ^↑^	18 2.0 ± 1.50 ^↑^	70 7.8 ± 1.64 ^↑^	11 1.4 ± 0.74 ^↑^	- -	256 28.4 ± 7.33 ^↑^

Data represent the sum (upper row) of chromosomal aberrations per group, and their mean ± SD (lower row) based on nine independent scorings. Significantly increased (*p* < 0.05) values are ↑—vs. all other experimental groups; I—vs. IRI; #—vs. all other samples, except AH + IRI; α—vs. 1 × STH + IRI sample; β—vs. 5 × STH + IRI sample; γ—vs. 10 × STH + IRI sample.

**Table 6:** Micronuclei (MNi), nuclear bud (NB), and nucleoplasmic bridge (NPB) formation after treatment with different combinations of cytotoxic drug irinotecan (IRI) with strawberry tree honey (STH) and artificial honey (AH). Single-IRI-treated cells and positive control (bleomycin—15 mg/L) cells were studied in parallel.

Group	Micronuclei (MNi)							Nuclear Buds (NBs)		Nucleoplasmic Bridges (NPBs)	
	Mean (MNi)_1000_ ± SD	Total (MNi)_9000_	Mean (BN_MN_)_1000_ ± SD	Total (BN_MN_)_9000_	Distribution of BN_MN_ Cells with			Mean (NBs)_1000_ ± SD	Total (NBs)_9000_ *	Mean (NPBs)_1000_ ± SD	Total (NPBs)_9000_ *
					1 MN	2 MN	3 MN				
**IRI**	13.0 ± 3.32 ^#^	117	12.0 ± 2.69 ^#^	108	100	7	1	7.3 ± 1.80 ^#^	66	0.7 ± 0.50 ^#^	6
**1 × STH + IRI**	6.9 ± 1.36	62	6.9 ± 1.36	62	62	0	0	3.9 ± 1.36	35	0	0
**5 × STH + IRI**	5.9 ± 0.93	53	5.9 ± 0.93	59	59	0	0	4.0 ± 1.12	36	0	0
**10 × STH + IRI**	7.4 ± 1.13	67	7.3 ± 1.00	66	65	1	0	4.4 ± 1.42	40	0	0
**AH + IRI**	12.1 ± 4.17 ^$^	109	11.8 ± 3.83 ^$^	106	103	3	0	7.8 ± 1.99 ^$^	70	0.1 ± 0.33	1
**Positive control**	17.6 ± 2.07 ^↑^	158	15.6 ± 1.81 ^↑^	140	123	16	1	9.8 ± 1.86 ^↑^	88	1.4 ± 0.73 ^↑^	13

Significantly increased (*p* < 0.05) values are ↑—vs. all other experimental groups; #—vs. all other samples, except AH + IRI and positive control; $—vs. all other samples, except IRI and positive control. *—BN cells contained only a single NB or NPB.

**Table 7:** Description of experimental groups and treatments. In vitro exposure of blood samples to the selected concentrations of all of the tested compounds.

EXPERIMENTAL DESIGN				
**Negative control** non-treated cells	**Treatments without cytotoxic drug**			
	Strawberry tree honey (STH)			Artificial honey (AH)
	1×	5×	10×	
	0.71 g/L	3.50 g/L	7.10 g/L	0.71 g/L
**Cytotoxic drug** Irinotecan (IRI)	**Combined treatments with** **cytotoxic drug**			
	1 × STH 0.71 g/L	5 × STH 3.50 g/L	10 × STH 7.10 g/L	AH 0.71 g/L
	IRI 9.0 mg/L	IRI 9.0 mg/L	IRI 9.0 mg/L	IRI 9.0 mg/L

Positive control sample was treated with 15.0 mg/L of bleomycin for 2 h, according to previous studies [57,58].

## Data Availability

The data presented in this study are available on request from the corresponding author.

## Supplementary Materials

The following supporting information can be downloaded at: https://www.mdpi.com/article/10.3390/ijms24031903/s1.
