# Supplementary material for: Protective Effects of Arbutus unedo L. Honey in the Alleviation of Irinotecan-Induced Cytogenetic Damage in Human Lymphocytes—An In Vitro Study
Source: Int J Mol Sci. 2023 Jan 18;24(3):1903. doi: 10.3390/ijms24031903 (PMC9916164; doi:10.3390/ijms24031903)
Supplement: Supplementary file 1 [file ijms-24-01903-s001.zip › ijms-2108468-supplementary.pdf]

Supplementary Materials: "Protective Effects of *Arbutus unedo* L. Honey in Alleviation of Irinotecan-Induced Cytogenetic Damage in Human Lymphocytes—In Vitro Study" (by Andreja Jurič, Irena Brčić Karačonji, Uroš Gašić, Dušanka Milojković Opsenica, Saša Prđun, Dragan Bubalo, Dražen Lušić, Nada Vahčić, and Nevenka Kopjar)

Table S1. Phytochemical composition of the strawberry tree honey (STH) used in the present study.

Descriptor	Amount
**Chemical marker of STH**	
Homogentisic acid (mg/kg)*	306.83
**Phenolic compounds (mg/kg)**	
Gallic acid	0.011
Protocatechuic acid	0.008
p-Hydroxybenzoic acid	0.138
p-Hydroxyphenylacetic acid	0.038
Caffeic acid	0.008
p-Coumaric acid	0.031
Ferulic acid	0.009
Quercetin	0.008
Apigenin	0.004
Chrysin	0.004
Pinocembrin	0.007
Acacetin	0.363
Hydroxymethylfurfural (mg/kg)**	29.0
Sugars (g/100 g)	
Fructose**	34.2
Glucose**	32.5
Sucrose**	<0.1
Diastase (DN)**	7.4
Water content (%)**	17.8
Electrical conductivity (mS/cm)**	0.583

*According to Brčić Karačonji and Jurica [5]; **according to Jurič et al. [12].
